# Evaluation of Toxic Properties of New Glycopeptide Flavancin on Rats

**DOI:** 10.3390/ph15060661

**Published:** 2022-05-25

**Authors:** Michael I. Treshchalin, Vasilisa A. Polozkova, Elena I. Moiseenko, Helen M. Treshalina, Andrey E. Shchekotikhin, Eleonora R. Pereverzeva

**Affiliations:** 1 Gause Institute of New Antibiotics, 11 B. Pirogovskaya Street, 119021 Moscow, Russia; vasilisa2006@gmail.com (V.A.P.); moiseenko.alena@gmail.com (E.I.M.); treshalina@yandex.ru (H.M.T.); shchekotikhin@gause-inst.ru (A.E.S.); pereverzeva-ella@yandex.ru (E.R.P.); 2 Organic Chemistry Department, Mendeleyev University of Chemical Technology of Russia, 9 Miusskaya Square, 125047 Moscow, Russia

**Keywords:** semi-synthetic glycopeptide antibiotics, vancomycin, eremomycin, flavancin, acute toxicity, chronic toxicity, rats

## Abstract

Glycopeptide antibiotics have side effects that limit their clinical use. In view of this, the development of glycopeptides with improved chemotherapeutic properties remains the main direction in the search for new antibacterial drugs. The objective of this study was to evaluate the toxicological characteristics of new semi-synthetic glycopeptide flavancin. Acute and chronic toxicity of antibiotic was evaluated in Wistar rats. The medium lethal dose (LD_50_) and the maximum tolerated doses (MTD) were calculated by the method of Litchfield and Wilcoxon. In the chronic toxicity study, the treatment regimen consisted of 15 daily intraperitoneal injections using two dosage levels: 6 and 10 mg/kg/day. Total doses were equivalent to MTD or LD_50_ of flavancin, respectively. The study included assessment of the body weight, hematological parameters, blood biochemical parameters, urinalysis, and pathomorphological evaluation of the internal organs. The results of the study demonstrated that no clinical-laboratory signs of toxicity were found after 15 daily injections of flavancin at a total dose close to the MTD or LD_50_. The pathomorphological study did not reveal any lesions on the organ structure of animals after low-dose administration of flavancin. Thus, flavancin favorably differs in terms of toxicological properties from the glycopeptides currently used in the clinic.

## 1. Introduction

**Figure 1:**
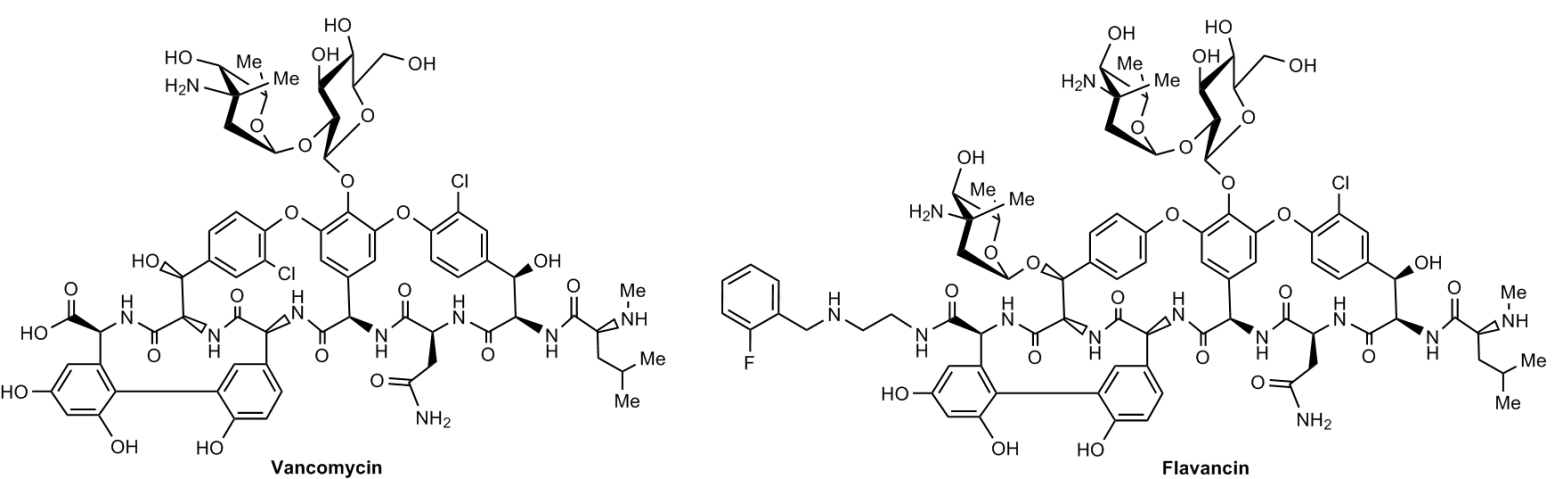
Chemical structure of vancomycin and flavancin.

Glycopeptide antibiotics, especially vancomycin (Figure 1), are generally drugs of choice for the treatment of infections caused by multi-resistant Gram-positive cocci (*Staphylococci, Streptococci* and *Enterococci* spp.) and rod-shaped (*Bacillus* and *Clostridia* spp.) bacteria. Because Gram-positive bacteria are characterized by high adaptability and the ability to form resistance to new antibacterial drugs, the development of new glycopeptides remains the main direction in the search for new antibacterial drugs for medicinal practice.

In the development of new antimicrobial drugs, the criteria for an “ideal” candidate as a second-generation glycopeptide with potential clinical value have been determined [1]. The spectrum of activity of such antibiotics should include Gram-positive microorganisms, including MRSA (Methicillin-resistant *S. aureus*), vancomycin-resistant enterococci (VRE), and glycopeptide-resistant *S. aureus*. The drug should also have a rapid dose-dependent bactericidal action and cause fewer side effects than vancomycin or teicoplanin.

In our previous study, a series of new amides of eremomycin was synthesized and developed [2]. Eremomycin is a glycopeptide antibiotic related to the vancomycin group of glycopeptides [3]. It has high antibacterial action against Gram-positive bacteria, including strains resistant to beta-lactams, tetracyclines, and fluoroquinolones [4]. It is 3–7 times more active than vancomycin (Table 1) [5]. Previous studies have shown that the disadvantage of eremomycin is its pronounced histamine-releasing activity [6]. This type of side effect of eremomycin did not allow approving it for use in a clinic.

In the novel series of derivatives, eremomycin was conjugated to a hydrophobic arylalkyl group via the carboxamide group [2,7]. This type of new eremomycin amides are more active than vancomycin against both glycopeptide-sensitive and glycopeptide-resistant Gram-positive strains. The highest activity among the new derivatives was shown by derivatives containing fluorobenzyl groups [2], of which the leader compound, *N*-(2-((o-fluorobenzyl)amino)ethyl)amide of eremomycin (flavancin, Figure 1), was chosen for in-depth preclinical evaluation. A study of its efficacy in a mouse model of staphylococcal sepsis showed that its ED_50_ is 7.5 times lower than that of vancomycin, and the therapeutic index is 317, which is 2.7 times higher than that of vancomycin [2].

The goal of this study was an in-depth evaluation of flavancin safety profile in the experiments on rats.

## 2. Results

### 2.1. Acute Toxicity

The quantitative parameters of acute toxicity of flavancin (MTD (LD_10_), LD_50_, and LD_100_ values) upon single intraperitoneal injection (IP) in rats as determined in this study are presented in Table 2.

After IP injection of high doses (170–300 mg/kg) of the drug, the animals died after 3–6 days. Drug administration in doses of 100–150 mg/kg led to the death of 0–3 animals after 7–10 days. There were no delayed deaths of the animals observed.

The macroscopical investigation of all animals euthanized on day 30 after the last death did not show any pathological changes related to the drug treatment. The mass coefficients of the internal organs (thymus, kidneys, spleen, liver, and heart) of these animals were similar to those of the control.

### 2.2. Chronic Toxicity

#### 2.2.1. Observations, Functional Indicators, and Clinical Laboratory Tests

There was no mortality from intraperitoneal injection of flavancin for daily doses 6 and 10 mg/kg within 15 days (total doses were equivalent to the MTD or LD_50_) and 30 days after finishing the course of drug administration in any of the experimental or control animal groups. There were no deviations in the behavioral reactions of the rats. The animals did not become restless, aggressive, or sluggish. Their muscle tone changed according to their age. There were no symptoms of bronchitis, conjunctivitis, or rhinitis. The drug application did not affect the skin, subcutaneous adipose tissue, hair, visible mucosa, or their consumption of food and water. Total count of leukocytes in blood of rats treated with flavancin did not differ from the control throughout the experiment (Table S1). The dynamics of the body weight change in treated groups did not differ from those in the control group. Overall, this indicates that flavancin did not have a pronounced general toxic effect in this chronic experiment.

On day 1 and day 30 post-treatment, the biochemical parameters of blood serum (Table S2), the electrocardiographic (ECG) parameters (Table S3), the weight indices of the internal organs of the rats, and the results of clinical urine analysis (Table S4) in both experimental groups were similar to those of the control group.

#### 2.2.2. Histological Evaluation

**Figure 2:**
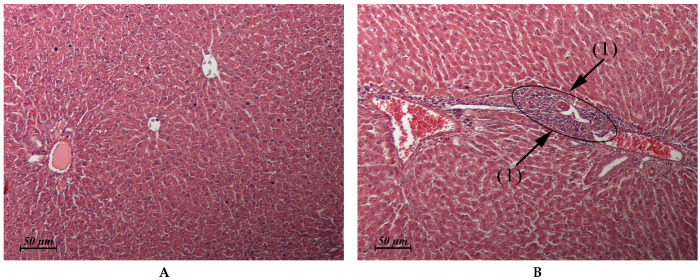
(**A**,**B**) Rat liver. (**A**) Untreated control. (**B**) Flavancin, 15 × 10 mg/kg/day, 1 day post-treatment. Micronecrotic focus in the vicinity of the triad (1) × 20.

**Figure 3:**
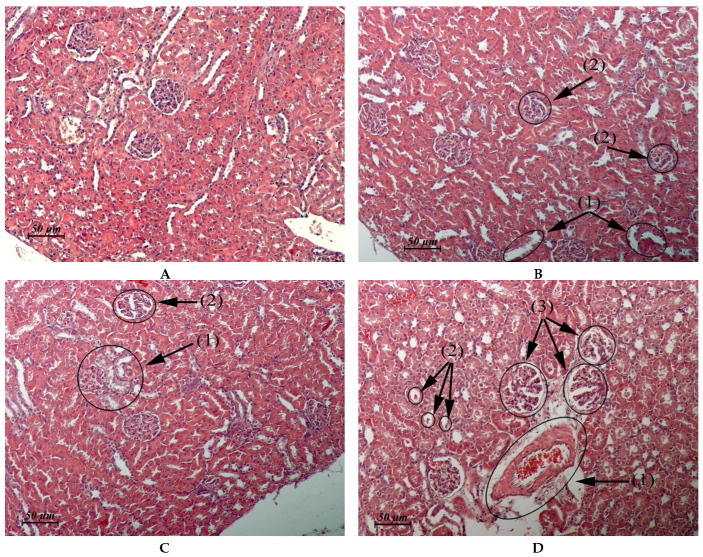
(**A**–**D**) Rat kidney. (**A**) Untreated control. (**B**) Cortical zone. Flavancin, 15 × 6 mg/kg/day, 1 day post-treatment. Vacuolar dystrophy of epithelium of some convoluted tubules (1). Thickening of the capsule and focal atrophy of the capillary network in glomerulus (2). (**C**) Cortical zone. Fuvancin, 15 × 10 mg/kg/day, 1 day post-treatment. Small focus of granular and vacuolar dystrophy of epithelium of convoluted tubules (1), clawed glomerulus (2). (**D**) Juxtamedullary zone. Flavancin, 15 × 10 mg/kg/day, 1 day post-treatment. Perivascular edema (1), destruction in the epithelial layer of some convoluted tubules (2), and clawed glomeruli with a sharply expanded lumen of the capsule (3) × 20.

**Figure 4:**
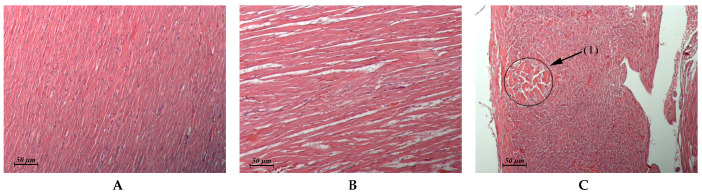
(**A**–**C**) Rat myocardium. (**A**) Untreated control. (**B**) Flavancin, 15 × 10 mg/kg/day, 1 day post-treatment. Interstitial edema. (**C**) Flavancin, 15 × 10 mg/kg/day, 1 day post-treatment. Focus of interstitial edema associated with toxic cardiomyopathy (1) × 20.

**Figure 5:**
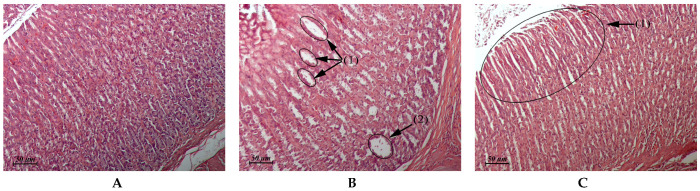
(**A**–**C**) Rat stomach. (**A**) Untreated control. (**B**) Flavancin, 15 × 10 mg/kg/day, 1 day post-treatment. Sharp expansion of the glands lumen (1) and cyst lined with squamous epithelial cells (2). The parietal cells prevailed in the glands. Destruction of parietal cells in single glands. (**C**) Flavancin, 15 × 10 mg/kg/day, 1 day post-treatment. Deep focal atrophy of the gastric gland epithelium and replacement of the glandular epithelium by the surface epithelium in this area (1) × 20.

The results of the pathomorphological study of the internal organs after flavancin administration are presented in Table 3 and in Figure 2, Figure 3, Figure 4 and Figure 5.

No pathological changes were observed in other organs.

Thus, the study of pathomorphological changes in the internal organs of rats under the influence of flavancin showed that the drug, applied 15 times in a single dose, which is 10 times higher than the ED_50_ (6 mg/kg = 1/15 MTD), does not have a damaging effect on the structure of most of the studied organs and tissues. Moderate pathological changes found in the kidneys immediately after the course of injections of the drug were completely repaired within a month. The drug administration at the total dose corresponding to LD_50_ resulted in damage to the structure of the liver, kidney, heart, and stomach. Although the structure of these organs is completely recovered within a month, it is necessary to take into account the possibility of their alteration with a significant overdose of the drug.

## 3. Discussion

The search for new glycopeptide antibiotics with improved chemotherapeutic properties is a current focus of antimicrobial drug development [1,8,9,10]. This is due to three main factors: the spreading of strain resistance to natural glycopeptides, restrictions for the indication of use due to their short half-life, rapid excretion, low accumulation in certain tissues, and the presence of side effects [11]. It is known that for this group of drugs, the most significant side effects are nephrotoxicity, ototoxicity, hematotoxicity, and anaphylactogenicity based on the development of pseudo-allergic reactions [12,13,14]. 

In the middle of the last decade, more effective antibacterial agents that are active against poly-resistant strains of Gram-positive pathogens were developed by major pharmaceutical companies. New semi-synthetic glycopeptides of the second generation (oritavancin, telavancin, dalbavancin) have been approved in the USA and Europe for the treatment of a limited range of infectious diseases. In their clinical use, new types of toxicity were discovered that are not characteristic of the first-generation of glycopeptides, such as an increased risk of developing osteomyelitis with oritavancin, nephrotoxicity, a risk of QT interval prolongation with telavancin, and an elevation of hepatic enzymes with dalbavancin [15,16,17].

Therefore, one of the current directions of our studies focused on finding new drug-candidates for the development of new semi-synthetic glycopeptides [2,7,18,19]. Particular attention is given to derivatives of the original antibiotic of this group, eremomycin, which was discovered by G.F. Gause et al. [3,20].

Some semisynthetic eremomycin amides have shown high activity in vitro against Gram-positive bacteria, including resistant strains for which the parent antibiotic was not active [21,22,23]. Eremomycin amides, such as the new semisynthetic glycopeptide eremomycin pirrolidide, exhibited higher therapeutic potential compared to the parent compound and less pronounced pseudoallergic reactions in animal tests [24]. These findings show that eremomycin amide derivatives are promising for the development of a new generation of glycopeptide antibiotics with improved efficiency and reduced adverse effects.

The new eremomycin aminoalkylamide flavancin showed certain advantages over the natural antibiotics vancomycin and eremomycin not only in terms of specific antibacterial activity but also in terms of their toxicological characteristics. No clinical laboratory signs of toxicity were found after 15 daily injections of flavancin at a total dose close to the MTD or LD_50_. The pathomorphological study did not reveal any lesions on the organ structure of experimental animals after low-dose administration of flavancin. Damage to the kidneys structure was expressed moderately on day 1 post-treatment and 30 days later, and the kidneys morphology was similar to that of the control. The leading side effects of vancomycin, namely acute tubulointerstitial nephritis and manifesting its nephrotoxicity [25], did not appear at all. No signs of hematotoxicity (neutropenia, thrombocytopenia) characteristics for vancomycin, namely hepatotoxicity, which is typical for dalbavancin, or cardiotoxicity, which is intrinsic to telavancin, were found [26,27,28]. In terms of toxic properties, flavancin is similar to eremomycin. There was no significant effects on the state of the peripheral blood, the coagulation system, or biochemical parameters, even when the doses recommended for humans were exceeded by 2–6 times and when the duration of administration was exceeded by 10–20 times in a chronic toxicity study of eremomycin in rats, dogs, and guinea pigs. The examination of the internal organ structure and function of animals treated with eremomycin showed that the drug can affect the kidneys and mucosa of the gastrointestinal tract, but these alterations are reversible [29]. Short-term pathologic changes in target organs (liver, kidneys, heart, and stomach) were detected only by morphological examination of the rats that received a high dose of flavancin. They did not reach critical values and almost completely disappeared a month after the end of the flavancin injection course.

Thus, according to our study results, flavancin favorably differs in toxicological properties from the parent compound eremomycin and glycopeptides currently used in the clinic.

## 4. Materials and Methods

A substance of flavancin, eremomycin *N*-(2((2-fluorobenzyl)amino)ethyl)amide hydrochloride, was synthesized following the previously reported method [2]. Its chemical structure is similar to that of vancomycin (Figure 1).

All experiments in vivo were performed in accordance with the European Convention for the Protection of Vertebrate Animals [30,31] and the National Standard of the Russian Federation R 53434-2009 “Good Laboratory Practice” [32]. The studies were carried out on adult Wistar rats, which were obtained from the animal breeding unit of the FMBA (Scientific Center for Biomedical Technologies of the Federal Biomedical Agency, Moscow, Russia). The animal studies were approved by the Ethics of Animal Experimentation of Gause Institute of New Antibiotics (Protocol No 03/2021).

### 4.1. Acute Toxicity

The acute toxicity study was performed on male (210–240 g) and female (180–220 g) Wistar rats. Animals were randomized into groups (*n* = 6) and received flavancin as a single intraperitoneal (IP) injection. The substance was dissolved in 5% glucose solution and administrated in doses of 100–300 mg/kg into the abdominal cavity of the rats. The concentration of flavancin in the injected solution was 10 mg/mL.

Acute toxicity was estimated by mortality, survival time, body weight gain/loss, food consumption, and clinical symptoms of intoxication, including behavioral reactions. Animals were observed for 30 days after the last death case, and then, the surviving animals were euthanized and subjected to necropsy for examination of internal abnormalities. The LD_50_ values and the maximum tolerated doses (MTD = LD_10_) were calculated by the method of Litchfield and Wilcoxon using StatPlus Professional 3.8.0 software [33].

### 4.2. Chronic Toxicity

Chronic toxicity was carried out on male (290–310 g) and female (220–240 g) Wistar rats, which were divided into groups (*n* = 10). The drug was dissolved in a 5% glucose solution and administered intraperitoneally daily for 15 days. The treatment regimen was 15 × 6 mg/kg and 15 × 10 mg/kg, with a 24 h interval between intraperitoneal administrations. Total doses were equivalent to the maximal tolerated dose (MTD) or medium lethal dose (LD_50_) of flavancin, respectively. Untreated animals were used as a control.

During the observation periods, the animals were followed up for any adverse effects. Body weight and food consumption were evaluated weekly. Hematological tests were performed using an automated hematology analyzer (Abacus Junior Vet, Diatron, Budapest, Hungary). Blood was withdrawn from the caudal vein before (day 0), during (days 7, 15), and after the drug administration course (days 3, 5, 7, 10, 20, and 30). The following parameters were measured: leukocyte count with differentiation type, erythrocyte count, hemoglobin, thrombocyte count, and hematocrit.

Clinical biochemistry parameters (alanine aminotransferase (ALT), aspartate aminotransferase (AST), alkaline phosphatase, creatinine, glucose, serum urea, bilirubin total and direct, total protein, and albumin) were determined automatically using a biochemical analyzer (ChemWell, Awareness Technology, Inc., Palm City, Florida, USA) on days 1 and 30 post-treatment. The urinalysis tests were carried out on days 1 and 30 post-treatment automatically using a Laura Smart analyzer (Lachema, Prague, Czech Republic). Electrocardiographic examination (second standard lead) was performed on days 1 and 30 post-treatment (electrocardiograph AKSION EK1E-07, Moscow, Russia).

The animals were euthanized on days 1 and 30 after the end of the drug administration course. All animals were subjected to necropsy. Thoracic and abdominal cavities and internal organs were inspected macroscopically. The organs were fixed in 10% neutral formalin, embedded in paraffin, and cut. Sections that were 5 µm thick were stained with hematoxylin–eosin. Statistical analysis was performed using Student’s *t*-test. Mean values and standard deviations were calculated for body weights, hematological parameters, and relative organ weights. The difference between the groups was considered significant at *p* ≤ 0.05.

## 5. Conclusions

Our evaluation of rats for flavancin subchronic toxicity upon parenteral administration demonstrated a favorable toxicity profile without side effects that are typical for the gold-standard vancomycin and glycopeptides currently used in the clinic. All side effects of flavancin were reversible within 30 days. Overall, the results of these studies suggest the high potential of flavancin as a novel antibacterial agent for the further drug development.

## Figures and Tables

**Table 1:** MIC values (µg/mL) of eremomycin and vancomycin [5].

Bacterial Strain	Eremomycin	Vancomycin
*Staphylococcus* *aureus ATCC 25,923*	0.13–0.25	1.0–1.0
*Staphylococcus epidermicus 533*	0.13–0.25	1.0–2.0
*Staphylococcus haemoliticus 602*	0.13–0.13	1.0–1.0
*Enteroccus faecalis 559*	0.25–0.25	1.0–1.0
*Enteroccus faecium 4*	0.25–0.25	1.0–1.0

Note: bacterial strain were obtained from the Museum of the Laboratory of Medical Microbiology of the State Scientific Center for Antibiotics (SSCA) Moscow, Russia.

**Table 2:** Quantitative parameters of acute toxicity of flavancin in rats (*n* = 6).

Parameter	Doses, mg/kg	
	Males	Females
LD_10_ (MTD)	94.8	98.1
LD_50_	157.7 (126.0 ÷ 188.0) *	159.4 (127.3 ÷ 190.0) *
LD_100_	236	242

Note: * Data shown as mean LD_50_ (significant deviation of LD_50_ for *p* ≤ 0.05). Abbreviations: LD_10_, 10% lethal dose; LD_50_, 50% lethal dose; LD_100_, 100% lethal dose; MTD, maximum tolerated dose.

**Table 3:** Pathomorphological findings after flavancin on days 1 and 30 post-treatment.

Organ	Day 1		Day 30	
	Flavancin of 6 mg/kg/day	Flavancin of 10 mg/kg/day	Flavancin of 6 mg/kg/day	Flavancin of 10 mg/kg/day
Liver (Control: Figure 2A)	Similar to control	Micronecrotic foci in the vicinity of single triads (Figure 2B)	Similar to control	Similar to control
Kidney (Control: Figure 3A)	Cortical and juxtamedullary zones: vacuolar dystrophy of the epithelium of some convoluted tubules. Thickening of the glomerular capsule and focal atrophy of the glomerular capillary network in single glomeruli (Figure 3B).	Cortical zone: small foci of granular and vacuolar dystrophy of the epithelium of some convoluted tubules in single-clawed glomeruli (Figure 3C). Juxtamedullary zone: moderate perivascular edema, multiple small foci of vacuolar dystrophy, single foci destruction in the epithelial layer of some convoluted tubules, clawed glomeruli with a sharply expanded lumen of the capsule (Figure 3D).	Similar to control	Similar to control. In one male, single glomeruli with a thickened capsule and single scarring glomeruli were found in the cortical and juxtamedullary zones.
Myocardium (Control: Figure 4A)	Similar to control	Foci of interstitial edema (Figure 4B). Some animals exhibited single foci of interstitial edema that was associated with toxic cardiomyopathy (Figure 4C)	Similar to control	Similar to control
Stomach (Control: Figure 5A)	Similar to control	Sharp expansion of the glands lumen of mucosa, which was sometimes accompanied by the formation of cysts lined with squamous or cuboidal epithelial cells. Parietal cells prevailed in the glands. The destruction of the parietal cells in single glands was observed (Figure 5B). We observed deep focal atrophy of the gastric gland epithelium with replacement of the glandular epithelium by the surface epithelium in these areas (Figure 5C).	Similar to control	Similar to control

## Data Availability

Data is contained within the article and its supporting information.

## Supplementary Materials

The following supporting information can be downloaded at: https://www.mdpi.com/article/10.3390/ph15060661/s1, Table S1: Total count of leukocytes (103 cells/mm^3^) in blood of rats treated with flavancin; Table S2: Clinical biochemistry parameters of blood serum of rats treated with flavancin; Table S3: Electrocardiographic parameters of rats treated with flavancin; Table S4: Clinical urine analysis of rats treated with flavancin.
