# Supplementary material for: Evaluation of Toxic Properties of New Glycopeptide Flavancin on Rats
Source: Pharmaceuticals (Basel). 2022 May 25;15(6):661. doi: 10.3390/ph15060661 (PMC9228439; doi:10.3390/ph15060661)
Supplement: Supplementary file 1 [file pharmaceuticals-15-00661-s001.zip › pharmaceuticals-1695064-supplementary.pdf]

Table S1 – Total count of leukocytes in blood of rats treated with flavancin

Groups	day during treatment		day post treatment				
	1	7	1	5	10	20	30
	male						
Σ LD_{50}	12.95 ± 4.05	12.22 ± 3.18	9.12 ± 1.73	11.43 ± 3.50	12.19 ± 3.92	11.54 ± 3.31	10.87 ± 3.90
Σ MTD	11.40 ± 4.05	12.48 ± 0.99	12.59 ± 2.81	9.41 ± 2.43	9.85 ± 4.85	11.47 ± 3.54	9.54 ± 3.65
Control	12.07 ± 3.73	12.06 ± 3.72	12.48 ± 3.17	11.52 ± 3.09	11.09 ± 2.87	13.02 ± 4.58	9.89 ± 3.31
	female						
Σ LD_{50}	11.99 ± 2.90	11.05 ± 2.76	14.57 ± 2.68	13.40 ± 2.85	10.58 ± 3.15	11.38 ± 3.95	12.58 ± 3.28
Σ MTD	10.80 ± 3.17	12.13 ± 2.55	11.08 ± 2.97	11.46 ± 3.21	12.17 ± 3.12	12.37 ± 3.31	13.04 ± 2.90
Control	11.96 ± 4.16	12.55 ± 1.74	12.19 ± 2.66	9.89 ± 1.45	11.53 ± 3.83	8.84 ± 4.02	11.24 ± 3.22

Table S2 – Clinical biochemistry parameters of blood serum of rats treated with flavancin intraperitoneally

Groups	ALT	AST	alkaline phosphatase	bilirubin total	bilirubin direct	creatinine	blood urea nitrogen	albumin	total protein
	day 1 post treatment								
Σ LD_{50}	117.3 ± 23.9	175.9 ± 9.5	112.4 ± 34.1	10.2 ± 0.8	4.1 ± 0.8	81.4 ± 10.9	9.8 ± 1.0	37.9 ± 1.6	71.7 ± 5.6
Σ MTD	100.5 ± 22.6	154.9 ± 17.7	111.3 ± 26.1	11.2 ± 1.6	5.6 ± 3.9	69.3 ± 4.1	8.1 ± 1.0	39.5 ± 1.1	72.5 ± 4.2
Control	111.1 ± 11.5	163.0 ± 12.6	104.7 ± 20.0	11.8 ± 0.8	4.9 ± 1.9	69.3 ± 8.7	7.1 ± 2.6	39.2 ± 1.6	73.5 ± 5.6
	day 30 post treatment								
Σ LD_{50}	87.8 ± 11.9	133.2 ± 15.6	103.1 ± 17.3	8.2 ± 1.5	2.2 ± 0.6	80.8 ± 6.2	6.1 ± 0.6	40.1 ± 1.5	76.4 ± 3.0
Σ MTD	108.5 ± 22.4	133.6 ± 15.8	117.8 ± 26.8	9.9 ± 0.8	2.5 ± 0.8	77.8 ± 16.2	7.2 ± 0.8	40.5 ± 1.4	73.2 ± 3.7
Control	111.9 ± 17.8	143.0 ± 33.2	131.2 ± 13.1	8.9 ± 0.7	2.5 ± 0.9	88.5 ± 8.8	5.2 ± 1.1	40.8 ± 1.1	77.8 ± 6.4

Table S3 – Electrocardiographic parameters of rats treated with flavancin intraperitoneally

Groups	day 1 post treatment				day 30 post treatment			
	Pulse rate/ min	QT/sec	RR/sec	QT %.	Pulse rate/ min	QT/sec	RR/sec	QT %
	male							
Control	394.15 ± 46.8	0.049 ± 0.01	0.152 ± 0.02	32.17 ± 4.12	378.42 ± 61.3	0.065 ± 0.02	0.158 ± 0.01	41.3 ± 5.4
Σ MTD	418.23 ± 38.3	0.052 ± 0.01	0.143 ± 0.01	36.3 ± 3.18	384.39 ± 43.7	0.071 ± 0.01	0.156 ± 0.01	45.6 ± 9.2
Σ LD_{50}	396.51 ± 43.9	0.063 ± 0.01	0.151 ± 0.03	42.02 ± 4.32	398.6 ± 37.7	0.066 ± 0.02	0.151 ± 0.02	43.8 ± 7.3
	femail							
Control	404.0 ± 13.5	0.049 ± 0.00	0.149 ± 0.01	32.5 ± 3.5	412.1 ± 11.8	0.049 ± 0.004	0.146 ± 0.01	33.4 ± 2.78
Σ MTD	378.6 ± 13.9	0.047 ± 0.004	0.159 ± 0.006	29.4 ± 3.0	396.3 ± 18.2	0.047 ± 0.002	0.152 ± 0.012	31.2 ± 3.7
Σ LD_{50}	412.2 ± 51.6	0.042 ± 0.007	0.147 ± 0.018	29.4 ± 6.9	407.5 ± 21.4	0.044 ± 0.003	0.147 ± 0.014	30.1 ± 2.4

Table S4 – Clinical urine analysis of rats treated with flavancin intraperitoneally

Groups	RBS	WBS	Bilirubin µmol/l	Urobilinogen µmol/L	Ketones mmol/L	glucose mmol/L	pH	Protein (g/l)	Specific weight
	day 1 post treatment								
	male								
Σ LD_{50}	negative	0–5	negative	negative	negative	negative	6.5 ± 0.6	0–0.3	1.025–1.03
Σ MTD	negative	0–5	negative	negative	negative	negative	6.7 ± 0.3	≤ 0.3	1.03–1.035
Control	negative	0–5	negative	negative	negative	negative	6.6 ± 0.4	≤ 0.3	1.02–1.025
	female								
Σ LD_{50}	negative	0–10	negative	negative	negative	negative	6.5 ± 0.3	0.3 – 0.5	1.025–1.029
Σ MTD	negative	0–5	negative	negative	negative	negative	6.4 ± 0.2	≤ 0.3	1.026–1.031
Control	negative	0–5	negative	negative	negative	negative	6.5 ± 0.2	≤ 0.3	1.02–1.027
	day 30 post treatment								
	male								
Σ LD_{50}	negative	0–5	negative	negative	negative	negative	6.6 ± 0.4	≤ 0.3	1.02–1.025
Σ MTD	negative	0–5	negative	negative	negative	negative	6.7 ± 0.2	≤ 0.3	1.015–1.02
Control	negative	0–5	negative	negative	negative	negative	6.7 ± 0.3	≤ 0.3	1.02–1.025
